# Supplementary material for: GViNC: an innovative framework for genome graph comparison reveals hidden patterns in the genetic diversity of human populations
Source: NAR Genom Bioinform. 2025 Sep 3;7(3):lqaf121. doi: 10.1093/nargab/lqaf121 (PMC12408910; doi:10.1093/nargab/lqaf121)

SUPPLEMENTARY INFORMATION

GViNC: An Innovative Framework for Genome Graph Comparison Reveals Hidden Patterns in the Genetic Diversity of Human Populations

Venkatesh Kamaraj[1,2], Ayam Gupta[1,2], Karthik Raman[1,2,3*], Manikandan Narayanan[1,2,4*], Himanshu Sinha[1,2,5*]

[1] *Centre for Integrative Biology and Systems Medicine (IBSE), Wadhwani School of Data Science and AI, Indian Institute of Technology (IIT) Madras, Chennai - 600 036, India*

[2] *Wadhwani School of Data Science and AI, IIT Madras, Chennai - 600 036, India*

[3] *Department of Data Science and AI, IIT Madras, Chennai - 600 036, India*

[4] *Department of Computer Science and Engineering, IIT Madras, Chennai - 600 036, India*

[5] *Department of Biotechnology, Bhupat and Jyoti Mehta School of Biosciences, IIT Madras, Chennai - 600 036, India*

**Co-corresponding authors: kraman@iitm.ac.in, nmanik@cse.iitm.ac.in, sinha@iitm.ac.in*

SUPPLEMENTARY TABLES

Table S1: Top ten bins with the most variability in the human pan-genome graphs. The 1KGP Genome Graph is abbreviated by ‘Complete’, and the Common_1KGP Genome Graph is abbreviated as ‘Common’. Variability is calculated as the count of variable nodes in the corresponding bin.

Genome Graph	Rank	Region	Variability	Remark
Complete	1	chr8:0-10 Mbp	523,905	Contains parts of the β-defensin gene clusters
Complete	2	chr16: 80-910 Mbp	492,481	Prone to structural variations and duplications
Complete	3	chr16:0-10 Mbp	477,967	Prone to structural variations and duplications
Complete	4	chr8:10-210 Mbp	457,413	Associated with an immune response (BLK)
Complete	5	chr7:0:110 Mbp	429,860	-
Complete	6	chr9:0:110 Mbp	426,628	-
Complete	7	chr4:0:110 Mbp	412,151	Associated with neurological development (HTT)
Complete	8	chr16:70:810 Mbp	400,705	Prone to structural variations and duplications
Complete	9	chr9:10-210 Mbp	396,851	-
Complete	10	chr3:0-110 Mbp	387,369	-

Common	1	chr6:30-410 Mbp	60,793	Overlaps with MHC (Major Histocompatibility Complex) region
Common	2	chr8:0-110 Mbp	54,037	Contains parts of the β-defensin gene clusters
Common	3	chr16:80-910 Mbp	49,302	Prone to structural variations and duplications
Common	4	chr8:10-210 Mbp	48,197	Associated with an immune response (BLK)
Common	5	chr16:0-110 Mbp	44,329	Prone to structural variations and duplications
Common	6	chr4:180-1910 Mbp	43,360	-
Common	7	chr7:0:110 Mbp	42,340	-
Common	8	chr3:0-110 Mbp	41,827	-
Common	9	chr9:0:110 Mbp	41,775	-
Common	10	chr4:0:110 Mbp	41,037	Associated with neurological development (HTT)

Table S2: Number of variants present in the HLA region for each population: These variants were used to create the population-specific HLA genome graphs.

Population	Number of Samples	Number of variants
AFR	662	91,683
AMR	347	78,676
EAS	504	77,743
EUR	503	77,132
SAS	489	76,253

Table S3: List of hypervariable nodes present in the population-specific HLA genome graphs: The genomic positions correspond to the coordinates in hg38. Yes denotes the presence of a hypervariable node for the particular genome graph at that genomic position, and No denotes the absence of the same.

Hypervariable Genomic Position	AFR HLA Genome Graph	AMR HLA Genome Graph	EAS HLA Genome Graph	EUR HLA Genome Graph	SAS HLA Genome Graph
29,699,787	Yes	No	No	Yes	No
29,767,771	Yes	Yes	No	Yes	Yes
29,951,575	Yes	Yes	No	Yes	No
29,991,587	No	Yes	Yes	Yes	No
30,182,970	Yes	Yes	No	No	No
30,605860	Yes	No	Yes	No	Yes
31,340,514	Yes	Yes	Yes	Yes	Yes
31,340,587	No	No	Yes	No	No
31,412,380	Yes	Yes	Yes	Yes	Yes
31,412,383	Yes	Yes	Yes	Yes	Yes
32,211,594	No	No	No	No	Yes
32,223,881	Yes	Yes	Yes	Yes	Yes
32,303,314	No	Yes	No	Yes	No
32,332,596	No	Yes	No	No	No
32,492,191	No	Yes	No	Yes	No
32,589,513	Yes	Yes	Yes	Yes	Yes
32,964,843	Yes	Yes	No	No	No
33,127,190	Yes	Yes	No	No	No
33,127,191	Yes	Yes	No	No	No

SUPPLEMENTARY FIGURES

Figure S1: Path-level representation of the most complex nodes in the 1KGP Genome Graph. Markers are placed on the edge that trace the reference path. The 12-degree nodes are present in chromosome 1, and the node IDs are *19298453 and 19298461*.

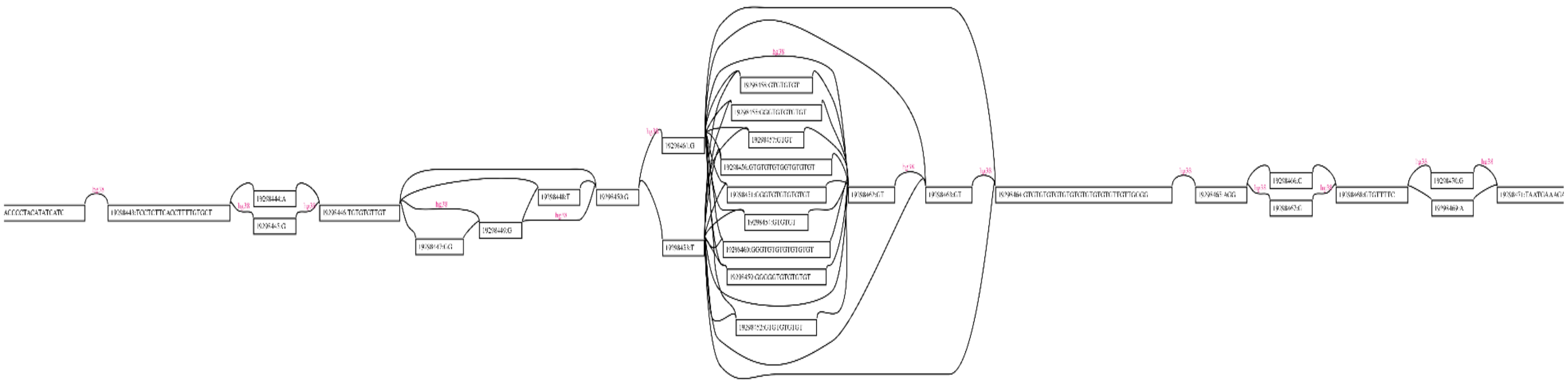

Figure S2: The median rate of recombination observed in each human chromosome.

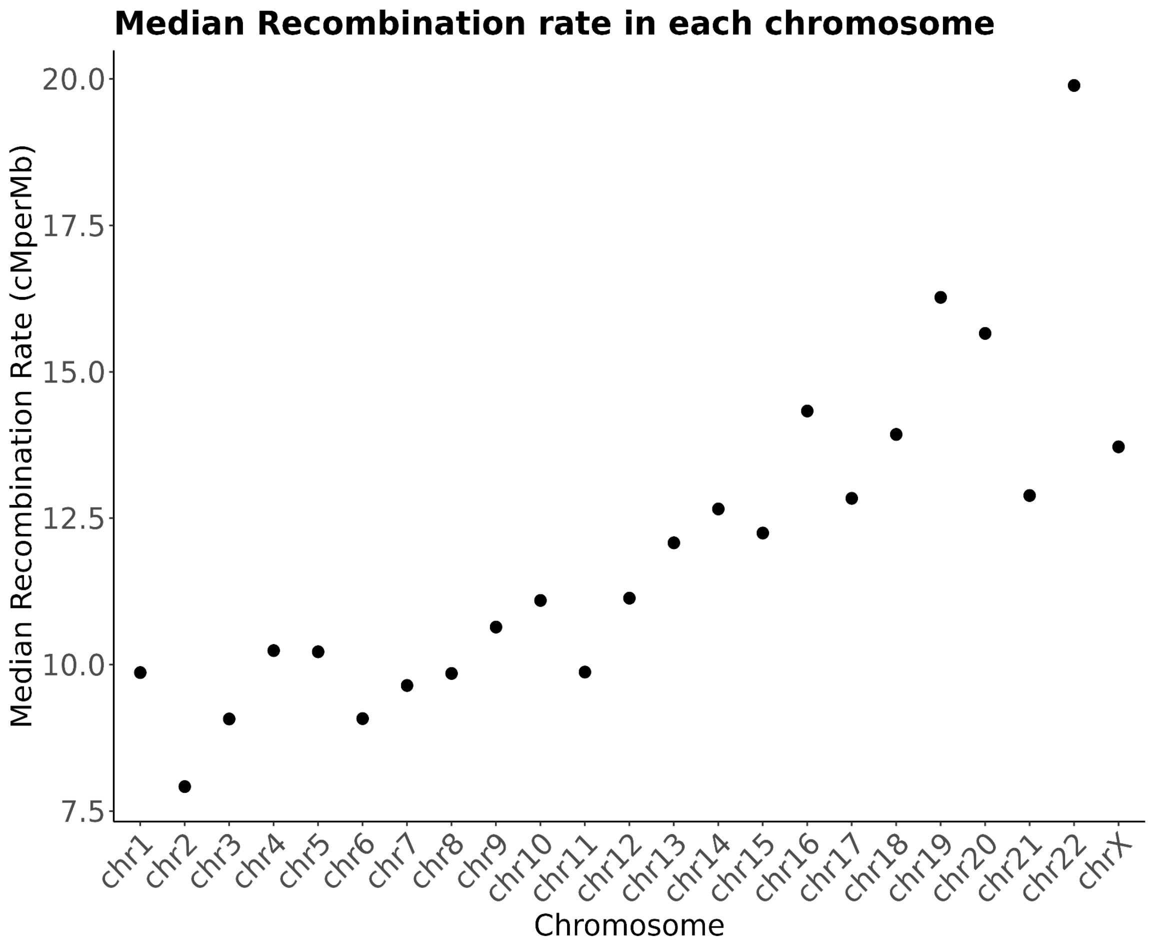

Supplement: lqaf121_Supplemental_File [file lqaf121_supplemental_file.pdf]
